# Lungworm Infection in Extensively Managed Goats in Nyangatom District of South Omo Zone, Southern Ethiopia

**DOI:** 10.3390/vetsci12050419

**Published:** 2025-04-28

**Authors:** Asrat Solomon Kenasew, Ayele Anjulo Kerkela, Tolisa Matiwos Tasisa

**Affiliations:** 1 Department of Veterinary Medicine, College of Veterinary Medicine and Animal Science, Jinka University, Jinka P.O. Box 165, Ethiopia; 2 Boreda Woreda Agriculture Office, Gamo Zone, Southern, Ethiopia; alefeya730@gmail.com; 3 Department of Clinical Study, School of Veterinary Medicine, Wollega University, Nekemte P.O. Box 395, Ethiopia; tolisamatiwos33@gmail.com

**Keywords:** *Dictyocaulus filaria*, *Mullerius capillaris*, pastoral area, prevalence, *Protostrongylus rufescens*

## Abstract

One of the common parasitic diseases of goats is lungworm infection, which causes significant economic losses due to losses in production and productivity. A cross-sectional study was carried out from December 2023 to November 2024 to determine the lungworm infection prevalence and associated risk factors in goats in the study area. Fecal examinations were conducted using the Baermann technique on the 384 goats selected by a simple random sampling technique. An overall prevalence of 33.85% was recorded, and *Dictyocaulus filaria* was the only lungworm identified during the study period. A statistically significant difference was observed between the prevalence of lungworms in female (45.65%) and male (23%) goats (*p* < 0.05). A higher prevalence (42.63%) was observed in younger goats than the prevalence (25.87%) in older goats, and the difference between the prevalence was statistically significant (*p* < 0.05). Regarding the body condition score, the difference was also statistically significant (*p* < 0.05). Non-dewormed goats were more prone to lungworm infection (41.24%) than dewormed goats (15.45%). Hence, lungworm infection is a considerable disease in the study area, and providing infrastructure for veterinary services was recommended.

## Simple Summary

Lungworm infection is one of the diseases of goats that causes a considerable loss of production. This study was conducted in pastoral areas where there is a lack of infrastructure and veterinary services. The study will help stakeholders intervene and conduct further research covering large pastoralist areas, and more attention should be given to these areas concerning their prevention and control.

## 1. Introduction

In many developing countries, livestock rearing/raising is one of the most important strategies to improve the living standards of the people [1]. Nearly two-thirds of the world’s livestock population is owned by developing countries; however, they produce less meat and milk, but have a much faster-growing human population than food production. Ethiopia is one of the countries in the world with the lowest production output. One of the factors responsible for low productivity is the poor health condition of its livestock [2]. Livestock production is a major component of the agrarian economy in developing countries and goes well beyond direct food production. Sales of livestock and their products provide immediate cash income to farmers and foreign exchange to the endowed countries [3]. Goat production is an important aspect of the country’s livestock farming and activity. Ethiopia has a diversified indigenous goat population of 52.5 million heads [4]. Despite the large goat population, the economic benefits remain marginal due to prevailing diseases, poor nutrition, poor animal production systems, reproductive inefficiency, management constraints, and lack of veterinary care [5,6].

The Nyangatom district, South Omo Zone, is one of Ethiopia’s pastoral areas with huge goat resources. In contrast to its huge goat population, the production and productivity, as well as economic yield from the sector, are very low due to different factors, including ailments affecting the livestock population [7]. Among these ailments that limit the economic returns from small ruminants like goats are many endoparasites, including lungworms [8].

The lungworms belong to Phylum Nemathelminthes, which has six classes, but only one of these, the nematode, contains worms of parasitic importance and are commonly called roundworms. They include genera of Ostertagia, Haemonchus, Trichostrongylus, Cooperia, Nematodirus, Dictyocaulus, Strongylus, Chabertia, Oesophagostomum, Stephanurus, Syngamus, Bonostomum, Muellerius, Ascaris, Toxocara, oxyuris, Spirocerca, Hebronema, Thelazia, Parafillaria, Trichuris, and Capillaria [9]. Available information indicates that the parasites occur in all ecological zones and production systems, and economic losses may be high due to clinical, chronic, and sub-clinical infections [10].

In ruminants, helminth infections are recognized as a major constraint to livestock production. Although infections are subclinical, they cause significant economic losses due to both mortality and reduced productivity of animals [11]. Parasitic infections pose a serious health threat and limit the productivity of livestock due to their associated morbidity and mortality [12].

Small ruminant lungworm parasites are extremely common, represent one of the biggest production bottlenecks, and are reported in many tropical and subtropical locations around the world. This is because the tropical and subtropical environments are ideal for their survival and growth [13]. They cause direct losses from deaths and indirect losses due to reduced productivity [14]. Control of these parasites is, therefore, essential for releasing the potential of goat production. For proper control, knowledge of parasitic diseases and their dynamics must be developed to establish rigid rules for their control, which are applicable to all regions. For these reasons, a study of the epidemiology of each parasitic disease should be limited to small areas. Therefore, to increase the potential of small ruminant production and to obtain the maximum benefits from them, the prevention and control of lungworms is essential. Environmental factors are conducive to lungworm infections, and it is considered an important disease [3]. The anti-parasitic pharmaceutical industries have made tremendous advancements in terms of developing new molecules with improved anthelminthic properties, but parasitism still prevails [15] due to the lack of veterinary care and infrastructure in pastoral areas, and the cost of the drugs themselves.

In some parts of Ethiopia, studies have been carried out on the prevalence of lungworms, but the pastoral areas, like the Benatsemay district, were neglected. Thus, the area incurs significant losses due to a lack of adequate knowledge of disease prevention and a lack of veterinary services. In these areas, the control of these parasitic helminths relies largely on the use of medicinal plants. Therefore, the current study was conducted to investigate the prevalence and to determine the associated risk factors of lungworms in the goats within the study area.

## 2. Materials and Methods

### 2.1. Description of the Study Area

The study period was from December 2023 to November 2024 in the Nyangatom district (Kangaten, capital city) of the South Omo Zone. The Nyangatom district is located between 4°85′–5°67′ N latitude and 35°75′–36°23′E longitude. The district is bordered by Dassenech Woreda in the south, Bench Maji and Salamago Woreda in the north, Hammer Woreda in the east, Kenya and South Sudan in the West, and has a total land area of 2652 km^2^. It is located 915 km southwest of Addis Ababa. The Nyangatom district is ecologically a lowland (kola/arid) with an altitude of 400–450 m above sea level. The district’s mean annual temperature ranges between 33 and 42 °C. The district has a mean annual rainfall ranging from 350 to 500 mm. The district has a livestock population of 676,215 cattle, 246,728 goats, 193,393 sheep, 77,419 poultry, and 24,171 donkeys [16].

### 2.2. Study Population

The source population consisted of goats residing in the selected kebeles of the Nyangatom district, coming to veterinary clinics, working, and resting places, and the study population was goats residing in the selected kebeles of the Nyangatom district.

### 2.3. Sampling Method and Sample Size Determination

The sampling procedure employed was a simple random sampling approach, and three rural kebeles were selected. The three kebeles selected for the study were Aipa, Nakereaman, and Narogoy. This was conducted based on their high population of goats when compared to other kebeles, which was believed to be representative of the district, as reported by the district agricultural office. Fecal samples were collected directly from the rectum of goats via simple random sampling. A desired absolute precision of 5% at a confidence level of 95% was used. Because no studies had been conducted in this area regarding this topic, a 50% prevalence was used. The sample size was determined using the equations given by [17], whereby n = z^2^ − pq/e^2^, where q = 1 − p, z = 1.96 e = precision error (0.05); p = expected prevalence of about 50%. Therefore, n = 1.96^2^ × (0.5) (1 − 0.5)/(0.05^2^) = 384.

### 2.4. Study Design and Methodology

A cross-sectional study design was used to determine the prevalence and associated risk factors of lungworms in goats in the Nyangatom district from December 2023 to November 2024.

### 2.5. Collection and Examination of Fecal Sample

From the selected animals, fecal samples were collected per rectum in a universal bottle. During the collection of the fecal samples, the following data (species of animal, study site, sex, age, body condition score (BCS), and deworming history) were recorded. The samples collected were transported to the Regional Veterinary Laboratory of Jinka within six hours of collection. Following [18,19], the technique used was the Baermann technique for the extraction of the lungworm larvae from the fecal samples. During the Baermann technique, a funnel fitted to a stand was taken. Then, a rubber tube was attached to the funnel with a clamp on the lower end. Then, the funnel was filled with lukewarm water. The feces were then wrapped to be examined for larvae with a double-layer gauze. The wrapped feces were kept on a tea strainer and it was lowered into the water in the funnel. After that, a beaker was kept under the funnel in case the rubber tube leaked. After 24 h, the clamp was opened and the aliquot was collected in a test tube. Then, the larvae were allowed to settle at the bottom of the test tube. After discarding the supernatant, it was microscopically checked for the presence of larvae [19]. As described by Taylor et al. [9], the identification of the species of lungworm encountered was carried out based on the characteristic morphological features as described. Accordingly, the posterior region of the first-stage larvae of *Dictyocaulus filaria*, *Protostrongylus rufescens*, and *Mullerius capillaris* were different. Moreover, when we observed grossly, their color was also different (*Dictyocaulus filaria*, *white*; *Protostrongylus rufescens*, *red*; and *Mullerius capillaris*, *grey-red*) [9].

### 2.6. Data Management and Analysis

The data collected were stored in a Microsoft Excel 2010 spreadsheet and analyzed using Stata Version 14. Descriptive statistics were used to summarize the data. The prevalence was calculated as the total infestation/infection cases divided by the total cattle examined. Univariable logistic regression was used to test the association between the prevalence of lungworm and the hypothesized risk factors. A *p*-value less than 0.05 at a 95 percent confidence level was considered when interpreting the results.

## 3. Results and Discussion

### 3.1. Prevalence of Lungworm

The overall prevalence of lungworm infection (*Dictyocaulus filaria* was the only lungworm observed) in the three study sites was 33.85% (130/384) in the study area. Its highest prevalence was observed in the Nekereaman study site, 14.58% (56/384), followed by Aipa, 9.89% (38/384). Narogoy was the study site with the lowest prevalence of lungworm infection, 9.38% (36/384) (Table 1).

### 3.2. Proportion of Sex, Age, Body Condition, and Study Site

Among the examined goats (*n* = 384) for lungworm infection, 52.08% were females and 47.92% were males. Among the examined goats, 28.65% were dewormed and 71.35% were non-dewormed.

The proportion of age was young, 0.476, and adult, 0.523, with a 95% confidence interval (CI) of [0.426–0.527, 0.473–0.574], respectively. The body condition scores were: good, 0.229; medium, 0.265; poor, 0.5, and a 95% CI of [0.190–0.275], [0.227–0.317, 0.449–0.550], respectively. The proportion of the study sites were: Aipa (0.254), Nekereaman (0.434), and Narogoy (0.312), with a 95% CI of [0.212–0.300], [0.384–0.484, 0.267–0.360], respectively.

**Figure 1:**
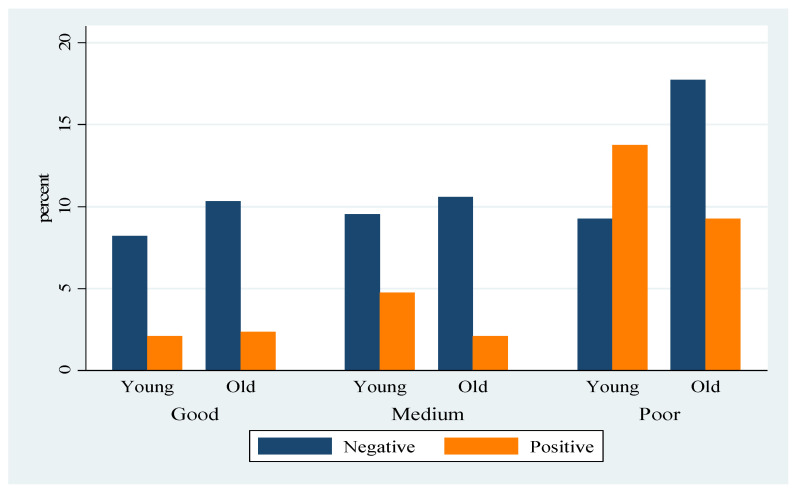
Prevalence of lungworm by age and body-condition groups.

The highest prevalence of lungworm infection was observed in young and poor body-conditioned goats. In good body-conditioned animals, a higher prevalence was observed in the older groups. But in the case of medium and poor body-conditioned goats, the younger ones were highly affected by lungworms (Figure 1).

The current study revealed that the prevalence of lungworm larvae in goats was 33.85% in the goats examined. The prevalence recorded in this study area is lower than the reports of the previous studies by [20], who reported 71.9% in Assela, and by [21], who reported 46.7% in the North Gondar Zone, Amhara region, and [22] who reported 79.4% in the South Omo Zone, Ethiopia. The lower prevalence in the current study could be attributed to the geographic variation. It might also be attributed to the method used for the detection of the larvae or the difference in the study area topography, which has a conducive environment for the survival of larvae [21] in another study area. According to Adem [23], the prevalence of small ruminant lungworms is different based on geographical and climatic factors of the spatial area. The current findings are comparable to the report of [23] (36.3%) in Edja Woreda, the Gurage Zone, central Ethiopia.

The current study reveals higher results than the report of [24], who reported a lungworm infection of 6.1% in and around Sebeta town, Ethiopia. Zerihun and Degefaw [13,25] also reported a lower prevalence of lungworm infection in goats (25.9%) in Mekelle and Bahirdar (28.7%), Ethiopia, respectively. The higher prevalence in the current study could be attributed to lower awareness among farmers of deworming their goats against lungworms and other helminthic infections. This was evidenced by the fact that more than 71% of the goats included in this study were not dewormed with anthelmintics. It could also be attributed to the lack of veterinary services and drug availability in the study area. It might also be attributed to the high contamination of the grazing pasture in the study area since communal grazing is prevalent in the Nyangatom district. The current study indicated that there was a higher prevalence of lungworm in female goats (45.65%) than in males (23%). This finding is similar to the reports of [23], and [26] in Ethiopia. In this study, young goats were affected more by lungworm (42.63%) than adults (25.87%), while poor body-conditioned goats were more prone to lungworm infection (44.84%) than others. This was also reported by [27] in Debra-Berhan town, Amhara region, Ethiopia, [28] around Wolaita town, and [21] in the North Gondar Zone, Amhara region. The prevalence rate of lungworm infection in young goats was also revealed by [29], who reported that 75.6% in young goats is significantly higher than that of adult goats (46.4%) in the Dale district, Southern Ethiopia. Recently, ref. [24] also reported similar findings in and around Sebeta, Ethiopia. This is because young ruminants in their first grazing season are clinically affected due to lower acquired immunity when compared with older animals, which have a strong acquired immunity [24,30]. The highest prevalence of lungworm infection in poor body-conditioned goats might be because the owners do not provide feed supplements, and the goats depend on natural grazing and browsing of the bush for feeding.

Regarding deworming history, a higher prevalence of lungworm infection was observed in non-dewormed goats (41.24%). This is because treatment with grazing management and its usage as a prophylactic treatment before the onset of the infective season is the most important method to control lungworm infection [30].

### 3.3. Risk Factor Analysis

Among the risk factors hypothesized, sex, age, BCS, and deworming history were found to be strongly associated with the prevalence of *D. filaria* infection (*p* < 0.05); however, the study site of the animals did not have a significant effect (*p* > 0.05) (Table 2).

*D. filaria* was the only species of lungworm identified in goats in the current study. The small lungworms, such as *Mullerius capillaris* and *Protostrongylus rufescens*, were not detected, which is consistent with the findings of [20,28]. The prevailing climatic conditions in the study area, which might not be conducive to the survival and breeding of the intermediate hosts, might be attributed to the absence of small lungworms [28]. In agreement with this finding, *D. filaria* as the predominant species circulating in Ethiopian goats managed under a traditional husbandry system was reported by [18,28]. Apart from Ethiopia, surveys in Iran [31] also reported *D. filaria* as the predominant lungworm in small ruminants, while in Norway [32] and Bulgaria [33], *M. capillaris* was the most prevalent species identified.

The current study indicated a lack of significant differences in the prevalence of lungworm among the study sites (*p* > 0.05). The sex of the animal was significantly associated with the prevalence of lungworm infection (OR (95% CI) = 2.581 (1.626–4.097), *p* < 0.05), which is in line with the report of Seifu [23] from Edja Woreda, the Gurage Zone, Central Ethiopia. But [13,28] reported a contrasting result. This difference might be because the resistance of female animals to lung infection can be reduced at the time of parturition and during early lactation. During the peri-parturient period and lactation, relaxation of the resistance of animals may result in female animals being unable to expel adult worms and cause a higher level of larvae [24].

The analysis of lungworm infection by the age of goats showed that the prevalence was significantly higher in younger goats than the older ones (OR (95% CI) = 0.470 (0.296–0.747), *p* < 0.05). The body condition score (OR (95% CI) = 2.030 (1.480–2.784), *p* < 0.05) and deworming history (OR (95% CI) = 2.030 (1.223–3.366), *p* < 0.05) were also statistically significantly associated with lungworm infection in goats, which is similar to the report of [23] in Edja Woreda, the Gurage Zone, Ref. [27] in Debra- Berhan town, Amhara region, Ethiopia, [28] in Wolaita Soddo and [25] in Bahirdar. In disagreement with this [24] reported that sex, age, body condition score, and deworming history were not significantly associated with lungworm infection.

## 4. Conclusions

In conclusion, the overall prevalence of lungworm infection in this study area was 33.85%. The only lungworm species identified from the studied goats during the study period was *D. filaria*. The sex, age, body condition score, and deworming history were among the risk factors that were significantly associated with the prevalence of lungworm infection. Hence, lungworm infection is a considerable disease in the study area, and it requires more effort from the stakeholders to prevent and control the disease. As a recommendation, scheduled regular and strategic deworming, supplementation of quality feed to goats, and further studies based on the PCR, postmortem, and fecal culture to accurately identify lungworm species were forwarded.

## Figures and Tables

**Table 1:** Prevalence of lungworm from Dec 2023 to Nov 2024 in the Nyangatom district.

Predicted Risk Factors	No. of Animals Examined	No. of Animals Positive	Prevalence (%)
Sex			
Male	200	46	23
Female	184	84	45.65
Age			
Young (<3 years)	183	78	42.63
Adult (>3 years)	201	52	25.87
Body condition			
Good	88	17	19.3
Medium	102	26	25.5
Poor	194	87	44.84
Study site			
Aipa	102	38	37.25
Nekereaman	164	56	34.15
Narogoy	118	36	30.5
Deworming history			
Dewormed	63	17	15.45
Not dewormed	191	113	41.24

**Table 2:** Logistic regression analysis of *D. filaria* infection in goats with different risk factors. OR: odds ratio; CI: confidence interval; BCS: body condition score; Ref: reference category.

Result	Std. Err.	Z	*p* > |z|	OR (95% CI)
Sex	0.608	4.02	0.000	2.581 (1.626–4.097)
Male				Ref
Female	0.630	4.09	0.000	2.649 (1.661–4.225)
Age	0.111	−3.19	0.001	0.470 (0.296–0.747)
Young				Ref
Adult	0.109	−3.27	0.001	0.459 (0.288–0.733)
BCS	0.327	4.39	0.000	2.030 (1.480–2.784)
Good				Ref
Medium	0.435	0.43	0.669	1.172(0.566–2.426)
Poor	1.145	3.89	0.000	3.530 (1.868–6.669)
Study site	0.1359	−0.86	0.392	0.875(0.645–1.187)
Aipa				Ref
Nekereaman	0.230	−0.76	0.445	0.804 (0.458–1.409)
Narogoy	0.241	−0.81	0.420	0.779(0.423–1.431)
Deworming history	0.5239	2.74	0.006	2.030(1.223–3.366)
Dewormed				Ref
Not dewormed	0.568	2.94	0.003	2.163 (1.292–3.619)
_cons	0.078	−4.16	0.000	0.203 (0.096–0.430)

## Data Availability

The original contributions presented in this study are included in the article. Further inquiries can be directed to the corresponding author due to privacy reasons.
